# P-1560. Risk Factors for Extended-Spectrum β-Lactamase-Producing *Escherichia coli* Urinary Tract Infections

**DOI:** 10.1093/ofid/ofae631.1727

**Published:** 2025-01-29

**Authors:** Julia Tellerman, Gabriella Khawly, Hsioa Che Looi, Jenna Dietz, Christopher J Myers, Rebecca Tsay, Savanah Russ, Ghinwa Dumyati

**Affiliations:** New York Emerging Infections Program, Rochester, New York; University of Rochester Medical Center, New York, New York; New York Rochester Emerging Infections Program at the University of Rochester Medical Center, Rochester, New York, Rochester, New York; Center for Community Health and Prevention, Rochester, New York; University of Rochester, Rochester, New York; New York Rochester Emerging Infections Program at the University of Rochester Medical Center, Rochester, New York; Center for Community Health & Prevention, University of Rochester Medical Center, Rochester, New York; New York Emerging Infections Program and University of Rochester Medical Center, Rochester, New York

## Abstract

**Background:**

*E. coli* is the leading cause of urinary tract infections (UTIs) worldwide. Antibiotic resistance among *E. coli* is increasing. We performed a case-control study to identify risk factors predictive of a positive extended-spectrum beta-lactamase (ESBL)-producing *E. coli* (ESBL-E) compared to a non-ESBL-E urine culture.

Table 1

**Figure:**
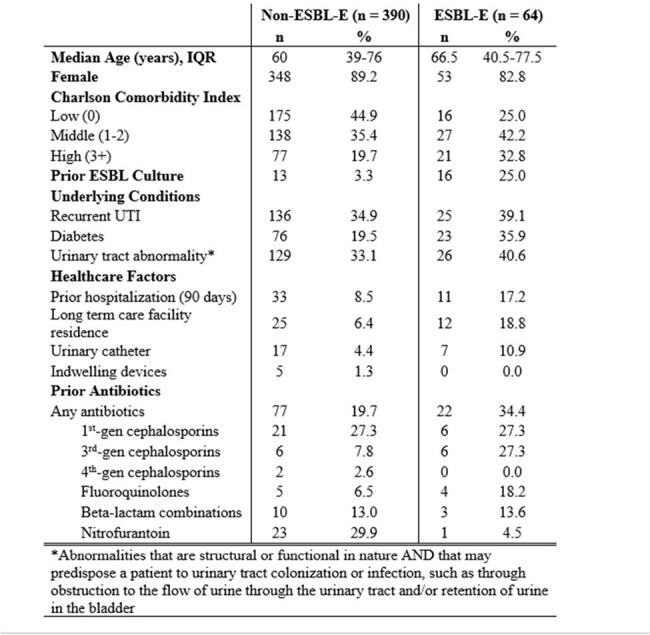

Demographic and clinical characteristics of patients with positive E. coli urine culture, Monroe County, August 2023

**Methods:**

Positive *E. coli* urine cultures were identified via laboratory and population-based surveillance in Monroe County as part of the CDC Emerging Infections Program in August 2023. Cases were defined as a county resident with a positive ESBL-E urine culture (resistant to ≥1 3rd-generation cephalosporin and nonresistant to carbapenems). Controls had a non-ESBL-E urine culture. All ESBL-E cases and a random sample of non-ESBL-E controls underwent medical record review to collect information on prior antimicrobial use, recent hospitalization, prior ESBL-E infection, underlying conditions, and prior long term care stay. Charlson Comorbidity Index (CCI) was calculated and categorized as low (0), middle (1-2), and high (3+). Multivariable logistic regression was used to identify independent risk factors.

**Results:**

We identified 2142 patients with an *E. coli* urine culture; 454 underwent chart review (64 ESBL-E, 390 non-ESBL-E). Patient characteristics are summarized in Table 1. Overall, patients were mostly female (88.3%) with a median age of 61 years (IQR 39-76). Cases more commonly received antibiotics in the 30 days prior to culture (34.4% vs. 19.7%, p = 0.007), had a hospitalization in the 90 days prior to culture (17.2% vs. 8.5%, p = 0.029), had prior ESBL-E culture (25.0% vs. 3.3%, p < 0.0001), a high CCI (32.8% vs. 19.7%, p = 0.006), prior LTC stay (18.8% vs. 6.4%, p = 0.0008), and diabetes (35.9% vs. 19.5%, p=0.003). After adjusting for confounding factors, only prior ESBL-E culture (OR = 8.22; 95% CI: 3.68-18.40) and diabetes (OR = 2.32; 95% CI: 1.31-4.09) were significantly predictive of the outcome.

**Conclusion:**

Patients with ESBL-E are more medically complex with increased healthcare exposure compared to patients with non-ESBL-E. Presence of a prior ESBL-E culture and diabetes were specific risk factors. Future studies are needed to better understand the collective impact of ESBL-E drivers to inform prevention strategies.

**Disclosures:**

**All Authors**: No reported disclosures

